# Effectiveness of Urea and Tolvaptan in the Treatment of Hypotonic Hyponatremia

**DOI:** 10.3390/jcm14103315

**Published:** 2025-05-09

**Authors:** Juan Delgado-Cuesta, Cristina Escorial-Moya, Antonio J. Vallejo-Vaz, Bernardo Santos-Ramos, Jose M. Varela, Enrique J. Calderón, Francisco J. Medrano

**Affiliations:** 1 Servicio de Medicina Interna, Hospital Universitario Virgen del Rocío, E-41013 Seville, Spain; juan.delgado.cuesta@gmail.com (J.D.-C.); cescorial@jesmo.net (C.E.-M.); jmvarelaa@gmail.com (J.M.V.); ecalderon@us.es (E.J.C.); 2 Departamento de Medicine, Facultad de Medicina, Universidad de Sevilla, E-41013 Seville, Spain; ajvallejo-ibis@us.es; 3 Grupo de Epidemiología Clínica y Riesgo Vascular, Instituto de Biomedicina de Sevilla, IBIS/Hospital Universitario Virgen del Rocío/CSIC/Universidad de Sevilla, E-41013 Seville, Spain; 4 Centro de Investigación Biomédica en Red de Epidemiología y Salud Pública (CIBERSP), E-28029 Madrid, Spain; 5 Servicio de Farmacia, Hospital Universitario Virgen del Rocío, E-41013 Seville, Spain; bernardo.santos.sspa@juntadeandalucia.es

**Keywords:** urea, tolvaptan, hypotonic hyponatremia, syndrome of inappropriate antidiuresis, heart failure

## Abstract

**Objective:** The objective of this study was to compare the effectiveness of urea and tolvaptan in the treatment of plasma sodium levels in patients with hypotonic hyponatremia. **Methods:** This was an observational, longitudinal, and retrospective study including all adult patients who received treatment with urea or tolvaptan for hypotonic hyponatremia from 1 April 2014 to 31 October 2023 at the Department of Internal Medicine, Virgen del Rocío University Hospital, Seville, Spain. **Results:** Forty-seven (55.3%) patients received urea and 38 (44.7%) tolvaptan. The drugs were prescribed for the treatment of syndrome of inappropriate antidiuresis (SIAD) in 59 (69.4%) patients. The mean blood sodium level at the start of treatment was 123.5 ± 6.2 mEq/L. Overall, 61.7% and 63.2% of patients treated with urea and tolvaptan, respectively, achieved a normal blood sodium level (*p* = 0.89), although the time to have their sodium levels corrected differed between both groups: 41.7 ± 76 days with urea and 21 ± 23.9 days with tolvaptan (*p* = 0.038). The following were significant in the multivariate study: Initial sodium value (*p* = 0.037), absolute sodium improvement (*p* = 0.041), and percentage sodium improvement (*p* = 0.033). Among patients with SIAD, 69.5% achieved a normal sodium level; this figure was 45.5% for patients with heart failure. Three patients reported side adverse events in the urea group and none in the tolvaptan group. **Conclusions:** Our data, reflecting real-world practice and follow-up of patients with hypotonic hyponatremia, suggest that both urea and tolvaptan are safe, well-tolerated, and have a similar effectiveness in correcting blood sodium levels in patients with hypotonic hyponatremia, overall and secondary to SIAD, though treatment with tolvaptan achieved this goal earlier than urea.

## 1. Introduction

Hyponatremia, defined as plasma levels of sodium below 135 meq/L, is the most common electrolyte disorder among both outpatient and inpatient individuals. Hyponatremia is estimated to affect approximately 5% of adults overall, 20% of individuals over 65 years of age, and 15% of hospitalized patients, and it has been associated with increased adverse outcomes, including a longer in-hospital stay, high use of resources, and high mortality [1,2]. Hyponatremia is more commonly the result of water retention that dilutes the sodium circulating in blood, thus decreasing the blood osmolality (the so-called hypotonic hyponatremia). Hypotonic hyponatremia promotes water entry into the brain, causing a cerebral edema responsible for many hyponatremia-related symptoms such as headache, nausea, confusion, lethargy, and depression, among others [3].

Hypotonic hyponatremia can be classified as hypervolemic, hypovolemic, and euvolemic. Hypervolemic hyponatremia can be found in disorders such as heart failure, liver cirrhosis, and nephrotic syndrome, among others. In these conditions, the effective arterial volume decreases, causing a non-osmotic stimulation of vasopressin secretion. On the other hand, hypovolemic hyponatremia is related to renal or extra-renal sodium loss. Finally, euvolemic hyponatremia is associated with the syndrome of inappropriate antidiuresis (SIAD), previously known as syndrome of inappropriate antidiuretic hormone (ADH) secretion (SIADH), or with excessive water intake conditions such as psychogenic polydipsia, primary polydipsia, or potomania [4].

Currently, there is limited evidence on the comparative efficacy of different treatments for hypotonic hyponatremia, e.g., for the treatment of SIAD or in heart failure, due to the challenges of conducting randomized clinical studies in this very heterogeneous population. Consequently, most clinical practice guidelines make their recommendations based on expert opinion consensus [5,6]. Among the strategies currently available for managing hypotonic hyponatremia are water restriction [7,8], tolvaptan [8,9,10,11], urea [12,13,14], and more recently sodium–potassium cotransporter inhibitors (SGLT2is) [15]. Tolvaptan and urea are currently considered the most effective drugs [2,5,6]. However, thus far, only two studies have compared the use of these drugs in patients with SIAD. One of them included only 12 patients and the drugs were not given in parallel groups but in a sequential manner where patients treated with satavaptan or tolvaptan were switched to urea [14], and the other concluded that tolvaptan was more effective and faster at normalizing sodium values than urea [16]. The evidence on managing patients with hyponatremia secondary to heart failure with these drugs is also rather limited. Recently, a retrospective observational study comparing 49 patients treated with urea with a control group of 47 subjects was reported. These authors found that 72.73% of those treated with urea normalized natremia levels versus 45.9% in the control group, with few adverse effects [17]. Therefore, studies comparing the efficacy and safety of both drugs in the treatment of patients with hypotonic hyponatremia are most needed.

The primary objective of our study was to compare the effectiveness of urea and tolvaptan in the treatment of plasma sodium levels in patients with hypotonic hyponatremia. Secondary objectives included assessing the factors that influence sodium levels in patients with hypotonic hyponatremia treated with either drug, the tolerance to these drugs, and the effectiveness in the correction of hyponatremia specifically in the subgroup of patients with SIAD.

## 2. Materials and Methods

We conducted an observational, longitudinal, and retrospective study, including all adult patients (≥18 years of age) who received treatment with urea or tolvaptan for hypotonic hyponatremia, either in an outpatient or in-hospital setting, from 1 April 2014 to 31 October 2023, at the Department of Internal Medicine, Virgen del Rocío University Hospital, Seville, Spain. Hypotonic hyponatremia was defined as a blood sodium level < 135 mEq/L with a blood osmolality < 272 mOsm/Kg. Patients who did not have any subsequent laboratory test after starting treatment with urea or tolvaptan were excluded from the study.

Since both urea and tolvaptan are hospital-restricted dispensed drugs in Spain, the patients receiving the drugs and the drug-related data were taken from the drug dispensing database of the Virgen del Rocío University Hospital pharmacy. Any other patient data were taken from the patients’ electronic health records. The urea drug dispensed in the hospital was Urea NM^®^ in 15 g sachets with powder for dissolution from the manufacturer Nutrición Médica SL (Madrid, Spain). For tolvapatan, the drug dispensed was the generic brand Tevagen^®^, in presentations of 30 and 90 mg tablets, from the manufacturer Teva (Tel Aviv, Israel). Both drugs are administered orally. Where required, alternative dosages of these drugs were prepared by the hospital pharmacy (e.g., tolvaptan 7.5, 15, or 45 mg).

There was no established protocol for managing SIAD in the hospital and clinical practice guidelines place urea and tolvaptan at the same level of recommendation. Therefore, the choice of drug and doses were made by each treating physician as per routine clinical practice and his/her own clinical experience. The type of drug used (urea or tolvaptan), along with their dose and length of treatment, were recorded. We did also collect other treatments prescribed, including water restriction and/or hypertonic saline (100 or 150 mL of hypertonic saline solution (NaCl 3%) over 20 min in a single dose the first day of treatment). When water intake restriction was prescribed, the patient and nursing staff were instructed that water intake should not exceed 1000 mL per day.

The baseline was defined as the time when the patient was prescribed treatment with urea or tolvaptan; therefore, laboratory tests are without treatment at this time point. Follow-up data included levels of natremia at 2–3, 7, 30, 90, 180, and 360 days, occurrence of side adverse events (SAEs), and type of SAE.

*Statistical analysis*: Qualitative variables are described as absolute (*n*) and relative (%) frequencies, and the Chi-square test (Fisher’s exact test where appropriate) was used for between-group comparisons. Quantitative variables were assessed for the normality of their distribution and are reported as mean ± standard deviation (SD) or median (interquartile range, IQR), as appropriate. The Student’s *t*-tests were used for between-group comparisons if the quantitative variables followed a normal distribution; Mann–Whitney U-tests were used otherwise. Those variables that had a *p*-value < 0.1 in the univariate analysis were included in the multivariate analysis. Tests were two-sided and statistical significance was set at *p* < 0.05. Analysis was performed using IBM SPSS statistical software (IBM Corporation, Somers, NY, USA), version 26.0.

*Ethics*: This study was approved by the Research Ethics Committee of the Virgen del Rocío University Hospital, Seville, Spain on 26 September 2024 [ref. FIS-TOL-2024-01] and conducted in accordance with the Helsinki Declaration, Good Clinical Practices, and relevant Spanish legal and regulatory requirements. Data were collected unidentified in a bespoke database designed for the present study. Participants’ data were coded when transferring data from the source databases/EHRs to the study database, to ensure data confidentiality.

## 3. Results

During the study period, 85 patients started treatment with urea or tolvaptan for the treatment of hypotonic hyponatremia. Forty-seven (55.3%) patients received urea and 38 (44.7%) tolvaptan. Thirty-five (41.2%) patients were male. The mean age of patients was 75.3 ± 12.6 years. The drug was prescribed for the treatment of SIAD in 59 (69.4%) patients, for the treatment of heart failure in 22 (25.9%) patients, and for the treatment of other causes of hypotonic hyponatremia in the remaining four (4.7%) cases. Among patients with SIAD, this syndrome was secondary to a drug in 16 (18.8%) cases, in 14 (16.5%) cases it was associated with a neoplasm, five (5.9%) cases were due to a benign respiratory pathology, and three (3.5%) cases were due to neurological pathology. In the remaining 26 (30.6%) patients the etiology of SIAD was unknown. Twenty-three (27.1%) patients also received hypertonic saline solution at the start of treatment, and 33 (38.8%) patients were prescribed water restriction. The mean blood sodium level at the start of treatment was 123.5 ± 6.2 mEq/L overall.

The baseline characteristics of patients stratified by the treatment who received (urea or tolvaptan) are shown in Table 1. The most common starting doses of urea and tolvaptan were 15 mg/day (34 [72.3%] cases) and 7.5 mg/day (19 [50%] patients), respectively. The mean age was similar in both groups; 47.4% of patients were male in the group treated with tolvaptan, versus 36.2% among those treated with urea. Patients in the group treated with tolvaptan had approximately 3 mEq/L lower baseline sodium level (121.9 ± 7.1 mEq/L) compared with patients treated with urea (124.9 ± 5.1 mEq/L), *p* = 0.046.

**Figure 1:**
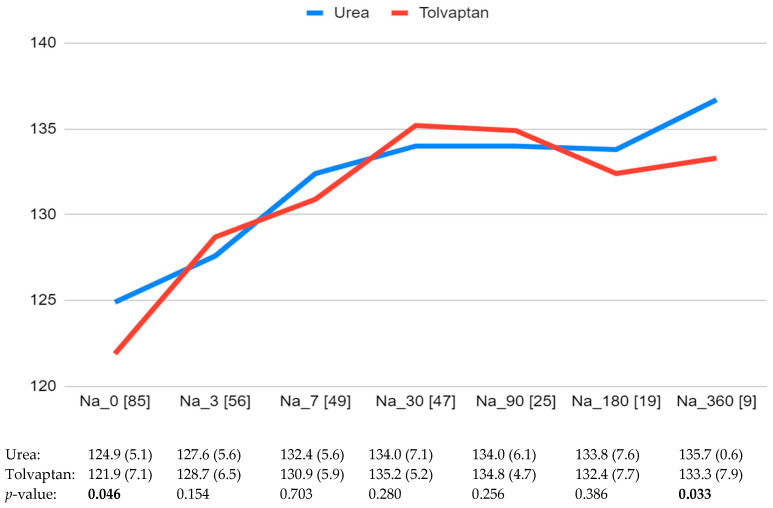
Blood sodium levels (mEq/L) at baseline and the different time points during the follow-up. The number of patients with sodium levels available at each time point is shown in brackets. Significant differences are indicated in bold.

The median follow-up time was 205 days (IQR 30-215). Sodium levels at the different time points over the follow-up based on the treatment received are shown in Figure 1. Parameters evaluating the changes in sodium levels over time are shown in Table 2. Overall, 61.7% and 63.2% of patients treated with urea and tolvaptan, respectively, achieved a normal blood sodium level (*p* = 0.89). The time to have their sodium levels corrected differed between both groups: 41.7 ± 76 days with urea and 21 ± 23.9 days with tolvaptan (*p* = 0.038). However, the time to highest Na level was similar: 30.2 ± 61.4 days with urea and 26.3 ± 34.6 days with tolvaptan (*p* = 0.542).

A multivariate analysis was also performed. Normalization of sodium levels was considered the dependent variable, and age, sex, treatment arm (urea or tolvaptan), water restriction, hypertonic saline solution infusion, and diagnosis were included as independent variables. None of them were statistically significant in the multivariate analysis.

The baseline characteristics of patients who achieved and who did not achieve a normal sodium level over the follow-up are shown in Table 3. Among patients with SIAD, 69.5% achieved a normal sodium level; this figure was 45.5% for patients with heart failure.

Table 4 and Table 5 show the baseline characteristics specifically of patients with SIAD and heart failure, respectively, who achieved and who did not achieve a normal sodium level over the follow-up.

Three patients reported SAEs in the group receiving urea, including nausea (two patients) and headache (one patient). Among these patients the treatment had to be discontinued in two of them, and the third one required the urea dose to be reduced by half. There were no reported SAEs in the group of patients receiving tolvaptan. Table 6 shows blood urea (mEq/L) and creatinine (mg/dL) levels at baseline and the different time points during the follow-up.

## 4. Discussion

To our knowledge, this is the first longitudinal real-world study comparing urea and tolvaptan for long-term treatment of hypotonic hyponatremia, a highly prevalent condition associated with high morbidity and mortality in clinical settings. In the study, including 47 patients treated with urea and 38 patients treated with tolvaptan as per routine clinical practice and followed-up over a median of 205 days, we did not find a significant difference between the two drugs in the proportion of patients achieving a normal blood sodium level over the follow-up (62.4% overall), suggesting that both drugs have a similar effect in attaining this goal. Although those patients treated with tolvaptan achieved this goal earlier, on average in half the time than patients treated with urea (21.6 vs. 41.7 days, respectively), the time to highest sodium was similar in both groups: 30.2 ± 61.4 days with urea and 26.3 ± 34.5 days with tolvaptan (*p* = 0.542). Additionally, as patients treated with tolvaptan started from somewhat lower sodium levels at baseline (121.9 mEq/L, vs. 124.9 mEq/L in the group treated with urea), this means that patients on tolvaptan achieved higher absolute and percentage increases in sodium levels compared with patients treated with urea (13.9 vs. 10.4 mEq/L and 11.8% vs. 8.5%, respectively). There was no difference in the groups treated with urea or tolvaptan in the percentage of patients prescribed initial concomitant therapy with hypertonic saline solution or water restriction. However, those patients who finally attained a normal sodium level had more frequently received any of those concomitant therapies compared with those patients who did not normalize their sodium levels, therefore suggesting that these therapies may help attain the treatment goal regardless of whether prescribing urea or tolvaptan.

From our study it is also noteworthy that although most patients achieved a normal sodium level with either treatment, still almost 40% of patients did not normalize their sodium levels, regardless of their treatment. This finding suggests the need for a better implementation of therapies (e.g., water restriction), the need to increase the doses of drugs, or the use of additional or alternative treatments (e.g., the most recent SGLT2i drugs may play a role) if we aim to improve the proportion of patients that obtain a correction of hypotonic hyponatremia.

Current European [5] and American [6] clinical practice guidelines consider water restriction a first-line measure in the treatment of hypotonic hyponatremia, as it can be implemented easily, though acknowledging a limited efficacy due to low patient adherence. Studies with water restriction of 1000 mL/day have shown a slight increase in plasma sodium levels, but still with a lack of response in 39% [7] to 55% of patients [8]. One possible explanation for the lack of response to fluid restriction in patients with hyponatremia is that some of the patients classified as having SIADH may actually have cerebral/renal salt wasting (C/RSW). C/RSW patients have identical clinical characteristics except for being hipovolemic and having appropriately increased ADH levels. In this way, they would not require water restriction but rather administer water and salt [18,19]. Water restriction was known to be applied in only 38.8% of participants in our study. Several factors may have contributed to this observation: in a retrospective study collecting data from clinical records such as ours, it is difficult to identify non-drug therapeutic recommendations such as water restriction when the treating physician does not make an explicit reference to it in the medical record. Another explanation is that the treating physician may have decided to start treatment directly using a second-line therapeutic strategy, such as the use of urea or tolvaptan, due to the challenges of effective water restriction in these patients or a lack of confidence in strategies that have demonstrated low efficacy.

The efficacy of tolvaptan in controlling hyponatremia was established in the SALT-1 and SALT-2 studies [9,10], showing an improvement in sodium levels compared to the placebo with few side effects. On the other hand, various observational studies exist with the use of urea, but to date, there is no randomized study on the use of this drug in SIAD. A retrospective study by Lockett et al. did not find any statistically significant differences in sodium levels 72 h after starting treatment with urea compared to water restriction [12]. Conversely, Martínez-González et al. [13], in a retrospective observational study of hospitalized patients with SIAD treated with urea, found that 44.6% of them achieved normal sodium levels.

However, to our knowledge, only one study has compared the use of urea and tolvaptan for long-term treatment in patients with SIAD so far; in this study, including only 12 patients, these drugs were given sequentially, where patients treated with a vaptan (satavaptan or tolvaptan) were later switched to urea; the authors found a good response in terms of sodium levels attained with both treatments and good tolerance to either drug [14]. In this way, Martinez-González et al. [16] have recently communicated a short-term comparative study of the effectiveness of tolvaptan versus urea in patients with SIAD. They concluded that tolvapan is more effective (83.7% vs. 59.8%) and faster in normalizing sodium values (4 ± 3.4 vs. 6 ± 3.6 days) than urea. Although in our study we also found that tolvaptan was faster than urea in normalizing natremia, we did not find differences in the effectiveness of both drugs. The difference between the two studies may be due to several reasons: a short follow-up time in the Martinez-Gonzalez paper that did not allow urea to achieve its maximum effect and that the mean basal sodium in the group of patients with urea was 2 mmol/L lower than in patients treated with tolvaptan.

The finding in our study that patients treated with tolvaptan achieved normal sodium levels earlier in the follow-up than those who received urea may be at least partly explained by the different mechanisms of action of both drugs. Urea is a hypertonic solute that is excreted in the glomerular filtrate, inducing osmotic diuresis and reducing natriuresis, resulting in increased free water clearance, whereas tolvaptan is a selective antagonist of the vasopressin type 2 receptor in the renal tubules which promotes the rapid excretion of free water without significantly affecting sodium excretion. Overcorrection or rapid correction of natremia in the first 24 h secondary to tolvaptan treatment is common; some real-world series have reported this issue in 12.1% to 24.6% of cases, with high morbidity and mortality related to central pontine myelinolysis due to osmotic demyelination [8,11]. In our study, the mean sodium increase in the first 48–72 h was 6.8 mEq/L, well below the 12 mEq/L in the first 24 h that is considered at risk for overcorrection, and no clinical signs of this condition were recorded in any case. The occurrence of overcorrection has been related to the use of high doses of tolvaptan; however, in our patients a low dose of tolvaptan was mostly used (the most common dose of tolvaptan was 7.5 mg/day).

During the follow-up, only three patients on urea reported SAEs: two with nausea and one with headache. In two of them, treatment was permanently discontinued, and in the third case the dose was halved, which resolved the side effect. Despite elevated uremia, no deterioration in renal function occurred during treatment with urea. No SAEs were reported with tolvaptan. These data support that both urea and tolvaptan, when managed appropriately, are safe and well-tolerated drugs, even in long treatments over time. A prior common problem with urea was its unpleasant taste, which led many patients to discontinue this treatment in the past. However, this issue has been resolved with new formulations that do not have this taste problem. In our study, no patient reported problems with the taste of the tablets.

The main cause of hypotonic hyponatremia in the patients included in our study was SIAD (69.4% of patients), followed by heart failure (25.9% of cases). Although urea and tolvaptan are considered second-line treatments after failure of water restriction in SIAD management guidelines [2,5,6], they are not mentioned or only briefly referenced in heart failure guidelines [20] or documents for patients with associated hyponatremia [1,21]. A recent retrospective observational study in HF compared 49 patients treated with urea with a control group of 47 subjects; the study found that 72.7% of those treated with urea attained normal sodium levels vs. 45.9% of patients in the control group, with few adverse effects [17]. In our study, 41 of 59 patients (69.5%) with SIAD normalized their sodium levels, but only 10 of the 22 patients (45.5%) with heart failure did so. Hyponatremia in patients with heart failure is hypervolemic, making it difficult to normalize sodium levels while this condition persists. Many patients with advanced heart failure remain hypervolemic, which may explain the lower response to urea and tolvaptan in this subgroup of patients.

In our study, both urea and tolvaptan showed a similar effectiveness in correcting hyponatremia in all patients overall and in those with SIAD, suggesting both drugs may represent appropriate options for managing hypotonic hyponatremia secondary to these conditions. The median time to peak natremia was greater than 30 days in both treatment arms in our long-term study and the effect was maintained over a mean follow-up of 205 days without the appearance of significant side effects. These data suggest that treatment for both heart failure-associated hyponatremia and uncorrected SIAD should be prolonged or even continued indefinitely. Further studies are needed to clarify this issue.

Some limitations should be acknowledged in our study. This study is observational and the results should be interpreted with caution as they are subject to confounding factors. Data on adherence to the study drugs during the follow-up were not available in the present study. Other limitations are typical of studies using EHRs and real-world data since the data are entered in health records by physicians as part of routine clinical care, as opposed to research purposes; therefore, the information recorded as well as its quality and completeness depends upon the accuracy of data recorded by the treating physicians.

## 5. Conclusions

Our data, reflecting real-world practice and follow-up of patients with hypotonic hyponatremia, suggest that both urea and tolvaptan are safe, well-tolerated, and have a similar effectiveness in correcting blood sodium levels in patients with hypotonic hyponatremia, overall and secondary to SIAD, though treatment with tolvaptan achieved this goal earlier than urea. A clinical trial comparing both therapeutic regimens is necessary to confirm these findings. Despite the use of either drug, more than one in three patients still remain with non-corrected sodium levels.

## Figures and Tables

**Table 1:** Baseline characteristics of patients treated with urea and tolvaptan.

	Urea (n = 47)	Tolvaptan (n = 38)	*p*-Value
Male, n (%)	17 (36.2%)	18 (47.4%)	0.297
Age (years)	77.0 (12.9)	73.3 (12.0)	0.586
Blood sodium levels, mEq/L	124.9 (5.1)	121.9 (7.1)	0.046
Creatinine, mg/dL	0.98 (0.44)	0.93 (0.55)	0.232
Urea, mg/dL	42.53 (28.35)	44.32 (38.84)	0.097
K, mEq/L	4.24 (0.71)	4.08 (0.77)	0.992
Diagnosis			
SIAD	31 (66.0%)	28 (73.7%)	0.626
Heart failure	13 (27.6%)	9 (23.7%)	
Others	3 (6.4%)	1 (2.6%)	
Concomitant treatment *			
Hypertonic saline solution	14 (29.8%)	9 (23.7%)	0.529
Water restriction	17 (36.2%)	16 (42.1%)	0.577

Qualitative data are shown as n (%); quantitative data are reported as means (standard deviations). SIAD, syndrome of inappropriate antidiuresis. * See Section 2 for details.

**Table 2:** Parameters evaluating the changes in sodium levels over the follow-up according to the treatment received.

	Urea (n = 47)	Tolvaptan (n = 38)	*p*-Value
Patients who achieved normal Na level	29 (61.7%)	24 (63.2%)	0.890
Time to achieve normal Na levels (days)	41.7 (76.0)	21.6 (23.9)	0.038
Highest Na levels (mEq/L)	135.3 (5.3)	135.8 (5.5)	0.744
Absolute change in Na levels (mEq/L)	10.4 (6.8)	13.9 (9.0)	0.053
Percentage change in Na levels (%)	8.5 (5.8)	11.8 (8.2)	0.031
Time to highest Na level (days)	30.2 (61.4)	26.3 (36.4)	0.542

Qualitative data are shown as n (%); quantitative data are reported as means (standard deviations). Na, blood sodium levels; normal Na levels: ≥135 mEq/L; time to achieve normal Na levels: time to first normal sodium value; change in Na levels: change in sodium level from baseline to the highest sodium level achieved.

**Table 3:** Baseline characteristics and treatment received among patients who achieved and who did not achieve a normal sodium level over the follow-up.

	Patients Who Achieved a Normal Na Level (n = 53)	Patients Who Did Not Achieve a Normal Na Level (n = 32)	*p*-Value
Male	19 (35.8%)	16 (50.0%)	0.202
Age (years)	74.8 (12.9)	76.2 (12.1)	0.747
Baseline Na level (mEq/L)	124.4 (6.5)	122.2 (5.5)	0.377
Diagnosis			
SIAD	41 (69.5%)	18 (30.5%)	0.241
Heart failure	10 (45.5%)	12 (54.5%)	
Others, n	2 (50.0%)	2 (50.0%)	
Concomitant treatment			
Hypertonic serum	12 (22.7%)	11 (34.4%)	0.241
Water restriction	22 (41.5%)	11 (34.4%)	0.516
Study treatment			0.891
Urea	29 (61.7%)	18 (38.3%)	
Tolvaptan	24 (63.2%)	14 (36.8%)	

Qualitative data are shown as n (%); quantitative data are reported as means (standard deviations). SIAD, syndrome of inappropriate antidiuresis; Na, blood sodium levels.

**Table 4:** Baseline characteristics and treatment received among patients with a diagnosis of syndrome of inappropriate antidiuresis (SIAD) who achieved and who did not achieve a normal sodium level over the follow-up.

	Patients Who Achieved a Normal Na Level (n = 53)	Patients Who Did Not Achieve a Normal Na level (n = 32)	*p*-Value
Male	19 (35.8%)	16 (50.0%)	0.202
Age (years)	74.8 (12.9)	76.2 (12.1)	0.747
Baseline Na level (mEq/L)	124.4 (6.5)	122.2 (5.5)	0.377
Diagnosis			
SIAD	41 (69.5%)	18 (30.5%)	0.241
Heart failure	10 (45.5%)	12 (54.5%)	
Others, n	2 (50.0%)	2 (50.0%)	
Concomitant treatment			
Hypertonic serum	12 (22.7%)	11 (34.4%)	0.241
Water restriction	22 (41.5%)	11 (34.4%)	0.516
Study treatment			
Urea	29 (61.7%)	18 (38.3%)	0.891
Tolvaptan	24 (63.2%)	14 (36.8%)	

Qualitative data are shown as n (%); quantitative data are reported as means (standard deviations). Na, blood sodium levels; SIAD, syndrome of inappropriate antidiuresis.

**Table 5:** Baseline characteristics and treatment received among patients with a diagnosis of heart failure (HF) who achieved and who did not achieve a normal sodium level over the follow-up.

	HF Patients Who Achieved a Normal Na Level (n = 10)	HF Patients Who Did Not Achieve a Normal Na Level (n = 12)	*p*-Value
Male	3 (30.0%)	6 (50.0%)	0.342
Age (years)	75.8 (13.2)	81.5 (13.1)	0.979
Baseline Na level (mEq/L)	126.5 (7.3)	123.3 (4.2)	0.104
Concomitant treatment			
Hypertonic serum	0 (0.0%)	2 (16.7%)	0.176
Water restriction	3 (30.0%)	1 (8.3%)	0.190
Study treatment			0.761
Urea	6 (46.2%)	7 (53.8%)	
Tolvaptan	4 (44.4%)	5 (55.6%)	

Qualitative data are shown as n (%); quantitative data are reported as means (standard deviations). Na, blood sodium levels; HF, heart failure.

**Table 6:** Blood urea (mEq/L) and creatinine (mg/dL) levels at baseline and the different time points during the follow-up.

	Urea (n = 47)	Tolvaptan (n = 38)	*p*-Value
Urea 0	42.53 (28.35)	44.32 (38.84)	0.097
Urea + 3 days	59.76 (44.81)	40.12 (37.85)	0.497
Urea + 7 days	72.41 (59.59)	45.56 (38.17)	0.080
Urea + 30 days	56.77 (31.41)	47.47 (38.38)	0.450
Urea + 90 days	59.00 (28.81)	71.00 (63.90)	0.022
Urea + 180 days	39.42 (15.70)	54.47 (57.96)	0.011
Urea + 360 days	43.00 (5.01)	50 (52.92)	0.049
Creatinine 0	0.98 (0.44)	0.93 (0.55)	0.232
Creatinine + 3 days	0.89 (0.40)	0.84 (0.57)	0.385
Creatinine + 7 days	1.02 (0.51)	1.01 (0.82)	0.165
Creatinine + 30 days	1.01 (0.51)	0.91 (0.58)	0.165
Creatinine + 90 days	1.07 (0.36)	1.47 (1.24)	0.002
Creatinine + 180 days	0.91 (0.30)	1.03 (0.67)	0.047
Creatinine + 360 days	0.96 (0.27)	0.85 (0.66)	0.274

## Data Availability

The data that support the findings of this study are available from the corresponding author [F.J.M.], upon reasonable request.
